# Towards the Crowdsourcing of Massive Smartphone Assisted-GPS Sensor Ground Observations for the Production of Digital Terrain Models

**DOI:** 10.3390/s18030898

**Published:** 2018-03-17

**Authors:** Ido Massad, Sagi Dalyot

**Affiliations:** Mapping and Geo-Information Engineering, The Technion, Haifa 3200003, Israel; sidomas@campus.technion.ac.il

**Keywords:** Digital Terrain Models, user-generated elevation data, Kalman filter, Assisted-GPS, ubiquitous mobile sensing

## Abstract

Digital Terrain Models (DTMs) used for the representation of the bare earth are produced from elevation data obtained using high-end mapping platforms and technologies. These require the handling of complex post-processing performed by authoritative and commercial mapping agencies. In this research, we aim to exploit user-generated data to produce DTMs by handling massive volumes of position and elevation data collected using ubiquitous smartphone devices equipped with Assisted-GPS sensors. As massive position and elevation data are collected passively and straightforwardly by pedestrians, cyclists, and drivers, it can be transformed into valuable topographic information. Specifically, in dense and concealed built and vegetated areas, where other technologies fail, handheld devices have an advantage. Still, Assisted-GPS measurements are not as accurate as high-end technologies, requiring pre- and post-processing of observations. We propose the development and implementation of a 2D Kalman filter and smoothing on the acquired crowdsourced observations for topographic representation production. When compared to an authoritative DTM, results obtained are very promising in producing good elevation values. Today, open-source mapping infrastructures, such as OpenStreetMap, rely primarily on the global authoritative SRTM (Shuttle Radar Topography Mission), which shows similar accuracy but inferior resolution when compared to the results obtained in this research. Accordingly, our crowdsourced methodology has the capacity for reliable topographic representation production that is based on ubiquitous volunteered user-generated data.

## 1. Introduction

A Digital Terrain Model (DTM) serves as the digital representation of the bare earth, usually in raster (grid) or vector-based Triangular Irregular Network (TIN) format. DTMs are used for a variety of applications, such as GIS, city modeling, land use studies, drainage control, and geology—to name a few. In the past, DTMs were almost exclusively produced via scanning and digitizing of contour maps. Nowadays, DTMs are often produced from data collected using high-end mapping platforms and technologies, such as LiDAR (Light Detection and Ranging), aerial and satellite photogrammetry, and InSAR (Interferometric Synthetic Aperture Radar). Regardless of the method used to produce the DTMs, most are produced by authoritative and commercial mapping agencies, such that the data collection process and post-processes tend to be costly and time-consuming, involving specialists in the field. Moreover, it is common that high-quality DTMs are not available in the public domain and must be purchased. There exist free open-source DTMs, such as ASTER (Advanced Spaceborne Thermal Emission and Reflection Radiometer) and SRTM (Shuttle Radar Topography Mission). These provide a representation having relatively low planar resolution (30 m), and with an elevation accuracy that is not uniform and consistent (approximately 10 m in 90% confidence level for most areas), while concealed areas near high vegetation and tall buildings, and areas showing extreme topographic variations (e.g., canyons) will show inferior accuracies [1].

The progress made in ubiquitous (noninvasive) data collection technologies, focusing on massive geographic data, have allowed the emergence of many applications, converting the collected data into usable geospatial infrastructure. Moreover, it allows the distribution of the derived information and knowledge to a wide number of users—commonly free of charge. These applications can vary from standard mapping platforms to more complex applications used for navigation and emergency situations. The employment of crowdsourced User-Generated Content (UGC) in general, and Volunteered Geographic Information (VGI) in particular, proves how widespread the use of user-generated geographic data and information have become. VGI is proven to be a fast, cost-effective, and reliable method in the production of mapping infrastructures, maintained on structured and reliable online mapping platforms and services [2]. Geographic user-generated data are mostly collected using ubiquitous handheld mobile devices and sensors (such as smartphones and tablets equipped with Assisted-GPS (A-GPS) capabilities), presenting both position and elevation data, and thus can be represented in different point models, e.g., trajectory vectors or TIN structure. The observed data have relatively a high Signal-to-Noise Ratio (SNR) that affects both on the positional accuracy and on the elevation accuracy. Taking this into account, handheld devices equipped with A-GPS technology can acquire position data having a relatively good accuracy that continues to improve (e.g., [3]). Furthermore, due to the sheer massive volume of the data, as well as modern post-processing techniques (e.g., filtering, noise-reduction), it has the potential to be (and is already proven to be) as reliable as authoritative data for various applications (e.g., [4]).

User-generated data, in particular, tend to contain more errors than equivalent data that is acquired in more traditional ways. These errors are hard to model and therefore are considered random errors (or random noise). The most common way of handling random noise is by applying a filter process, whereas such filter is usually implemented in a convolution process and can operate both in 1D and in 2D. The most common filter used for DTM production is the low-pass filter, although other types of filters are also applicable and can be considered (depending on the type of data acquired), such as multiple regression, gradient type, 2D Gaussian, 2D singular spectrum, and Kalman. In addition to a random noise filter, gross error detector should also be applied. This type of detector is usually simpler to implement than noise filters, as gross errors are easier to model and detect than random noise and are also less frequent. Kalman filter is proved to be effective in many GPS related technologies, such as navigation and guidance, such that it can be regarded as best fit for the implementation of noise reduction of massive A-GPS user-generated observations. The filtering process is an algorithm that uses a series of measurements observed over time, which contain random noise among other inaccuracies and produces estimates that are usually more accurate than a single measurement.

This research paper will demonstrate the feasibility of producing a reliable DTM infrastructure based solely on massive crowdsourced user-generated A-GPS observations from ubiquitous mobile sensors. Among others, this research paper will focus on data quality assessments of specific observations parameters, shaping the data filtering process implemented specifically for A-GPS data type, to generate a uniform and homogenous DTM infrastructure. Here, the analysis and assessment of two-dimensional Kalman filter algorithm for this task are investigated, together with the statistical analyses for the examination of the accuracy of the generated DTMs in respect to authoritative and SRTM DTMs.

## 2. Related Research

Although aerial photogrammetry and LiDAR can produce DTMs having high planar resolution (~1–3 m) and high accuracy (less than 1 m)—both on the vertical and horizontal planes—quality issues remain in some environments, such as dense residential areas, near tall buildings or in dense vegetation areas [5,6]. The ground survey, on the other hand, can offer similar-to-higher accuracy and resolution values with lesser post-processing requirement; still, it suffers from tedious field work that requires being present during the observation stage. The use of handheld mobile devices equipped with A-GPS sensor capabilities presents a very similar working process to the ground survey via passive ubiquitous observations. Still, it offers medium positional accuracy of observations due to relatively high SNR, and the fact that it uses reduced data-channels received from the GNSS satellites (e.g., [7,8]). Hence, the implementation of crowdsourcing methodologies on the user-generated data seems logical. Still, to this date, almost all geographic user-generated tools and applications focused exclusively on data in the horizontal plane (2D data), and there is very little research dealing with geographic UGC that is related to the vertical (elevation) dimension. Research is mostly concerned with 3D visualization of the surface in open-source mapping platforms, such as OpenStreetMap (OSM) [9], and the generation of coarse 3D building models [10], whereas most applications use elevation data provided from external sources that are not user-generated in nature, such as Bing Maps and SRTM. Thus far, almost all 3D data in OSM is related to data keys that users insert and edit manually when providing data—and not directly from observations. The best example is the mapping of buildings, where the observations themselves are provided in 2D, while a supplementary elevation key is inserted for height above the ground (as oppose to elevation), and is rarely applied to most types of OSM features: only 0.003% of all OSM nodes, for example, have the key elevation, while only 1.4% of OSM building features that exist in OSM have the key height [10]. Such that only rarely elevation data is retrieved from OSM, while it is mainly used for planar information integrated with other topographic sources, such as SRTM and ASTER (e.g., [1]).

A-GPS sensor capability implemented in mobile handheld devices has changed much in the last decade and can provide today an absolute planar accuracy in outdoor environments of 5–8.5 m horizontally, and 6–12.5 m vertically (e.g., [3,11,12]). This is achieved thanks to the hybrid locational system procedure that is nowadays known as “the seven technology enablers”: A-GPS, massively parallel correlation, high sensitivity, coarse-time navigation, low time-of-week, host-based GPS, and RF-CMOS. Further accuracy improvements can be achieved by integrating, and thus enriching, A-GPS observations with other types of built-in sensor readings existing on smartphones, such as a gyroscope, an accelerometer, a compass [13,14], a barometer [15], Wi-Fi [16], and reference maps [17]. Thus far, geographic user-generated platforms (mainly OSM) presented poor support for topographic data—although it is directly collected by the users—retrieving this data from external sources.

The improvement in new technologies used for user tracking via smartphones yields various studies that focus on exploiting this potential. In healthcare, for example, researchers proved how tracking the behavior of people by using data collected from various ubiquitous sensors embedded in smartphones (MEMS) can significantly improve the tracking of users’ physical state, considerably reducing the response time of health services [18]. Transportation studies showed how using the location data retrieved from smartphones can be used to accurately identify and extract the physical locations of road lanes, which are required for autonomous vehicles’ navigation [19], or the capacity to accurately classify different transportation modes by fusing readings from the embedded smartphone sensors [20], and also to better plan navigation routes [21]. Other studies also aimed to make use of the elevation data retrieved from smartphones’ location data, showing how it can be used to assess road roughness [22], or detect roads that are elevated from the ground [23], proving that height data can also be used. Still, no research aimed to show the potential of these technologies in producing a topographic representation, combined with the employment of crowdsourcing methodologies.

Filtering processes are commonly used to reduce errors, mainly random ones, which are associated with the high-frequency component of the data. Low-pass filter is perhaps the most commonly used for DTM production [24], although other methods are also employed, such as multiple regression with its variants [25], 2D Gaussian filter [26], morphological filter [27], 2D singular spectrum filter [28], and Kalman filter with its variants [29]. Using two-dimensional Kalman filter on DTMs to reduce random errors [30] is made by using a prediction model based on Euler method, i.e., making use of the previous point’s slope value and the distance between points to predict the next point value. Still, the main weakness exists in the boundary conditions, since Kalman filter works over time: the further it advances, the more accurate its estimation becomes. Since it might skew the results depending on the starting position of the process, the filter can be implemented simultaneously from different ends (boundaries), later merging the results into one cohesive outcome [31].

## 3. Methodology

The algorithms developed in this research rely entirely on ubiquitous massive crowdsourced position attributed data, namely ellipsoidal latitude, longitude, and elevation, together with the horizontal accuracy value calculated by the embedded A-GPS sensor. The DTM generation workflow is performed in five consecutive phases: (1) data collection; (2) pre-processing; (3) grid construction; (4) Kalman filter implementation—filter and smoother; and, (5) conversion of results from ellipsoid elevation to geoid elevation.

### 3.1. Data Collection

**Figure 1:**
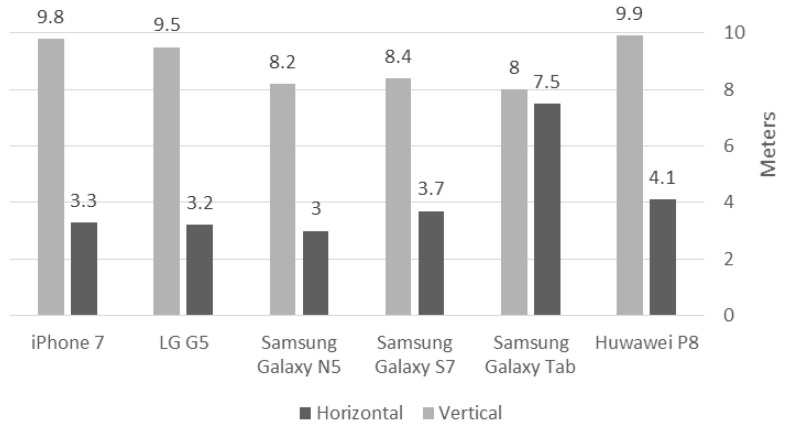
Smartphone model’s accuracy test results: grey bars depict the horizontal accuracy; black bars depict the vertical accuracy.

Since most mobile devices today are equipped with A-GPS technology, we aimed to collect position and elevation data in the experiments with various mobile smartphone devices. For the Android phones, the app used for collecting the data is GPS Logger (https://code.mendhak.com/gpslogger), and for the iOS Phone, the app used is SensorLog (https://itunes.apple.com/us/app/sensorlog/id388014573). Both apps collect longitude, latitude, altitude, speed, direction (last two are calculated from observations), number of observed satellites, horizontal accuracy, time, and date. The need for installing apps prior to collecting data is required, which to some extent contradicts the idea of ubiquities data collection. Still, the focus of this research is to prove the feasibility of producing qualitative topography representation from A-GPS data using the 2D Kalman filter as a fundamental stage, before using location data that is available on the Internet, such as OSM. Test areas are chosen such that they will give a good variety of different environmental settings and conditions to facilitate more robust analyses of various existing collection scenarios (described in the next Section). To attain a crowdsourced-like experiment, participating users were given a variety of smartphone device models, which were first examined to validate that their observation accuracy, both for the horizontal and vertical dimensions, are reliable in terms of current research in the field. Since the apps store metadata for the GPS observations, in case the data is collected from inferior mobile devices that have low accuracy levels, these can be filtered from the accumulated observation before continuing to the next processing stage. Figure 1 depicts the average accuracies achieved in our examinations, both for static and dynamic observations, showing that accuracy values are in line with the typical A-GPS current accuracies. It should be noted that in our field campaigns (Section 4), we assumed that the different mobile devices deliver similar raw observation quality, meaning that the 2D Kalman filter parameters were not adjusted to handle each device separately.

### 3.2. Pre-Processing

Although Kalman filter is reliable for filtering gross errors, a preliminary filtering process is performed on the raw collected data to remove any observations suspected unsuitable to use. These are mainly observations having low horizontal accuracy values, as measured by the device (app), as well as observations having no elevation value (e.g., observations measured during high PDOP phase). Transformation of the raw coordinate observations represented in Geographic system {ϕ, λ, h} (Ellipsoid WGS’84, EPSG:4326) to the local Cartesian system (*N*, *E*, *U*) of the new Israel Transverse Mercator (ITM, EPSG:2039) projection is implemented via a 7-parameters transformation, depicted in Equation (1). Observations are transformed to allow analysis of the results with respect to the authoritative reference DTM given in ITM.

(1) $$\left[\begin{array}{c} N\\ E\\ U \end{array}\right]=\left[\begin{array}{c} c_{x}\\ c_{y}\\ c_{z} \end{array}\right]+(1+s)\cdot \left[\begin{array}{ccc} 1 & -r_{z} & r_{y}\\ r_{z} & 1 & -r_{x}\\ -r_{y} & r_{x} & 1 \end{array}\right]\cdot \left[\begin{array}{c} X\\ Y\\ Z \end{array}\right]$$

where [*X*, *Y*, *Z*] are the raw measured coordinates value in geocentric system, [*c_x_*, *c_y_*, *c_z_*] are the coordinates value of reference point in the Cartesian system, *s* is the scale factor, [*r_x_*, *r_y_*, *r_z_*] are the rotation angles, and [*N*, *E*, *U*] are the coordinates value transformation results in ITM.

### 3.3. Grid Construction

The developed two-dimensional Kalman filter operates on two orthogonal axes simultaneously. Since location data is collected sporadically and irregularly, the transformed point locations have arbitrary and dispersed point positions data-structure unsuitable for the implementation of the two-dimensional Kalman filter. To solve this, a grid construction is performed. Since observations are mostly dense, presenting in our experiments localized areas, it was decided to use the Inverse Distance Weighted (IDW) interpolation method, which is also commonly used to generate regularly spaced DTMs [32,33]. IDW, considered a deterministic and local interpolation method, determines an elevation value of a point *p* by using a distance-weight calculation from other points within a predefined neighborhood [34]. IDW method used here consists of point height determination that is based on a minimum of 12 point neighbors within a radius of 250 m while using a distance with an exponent value of 2. The planar resolution of the grid can be chosen as required; the assumption is that the higher the resolution is, the more accurate the Kalman estimation should become.

### 3.4. Kalman Filter

**Figure 2:**
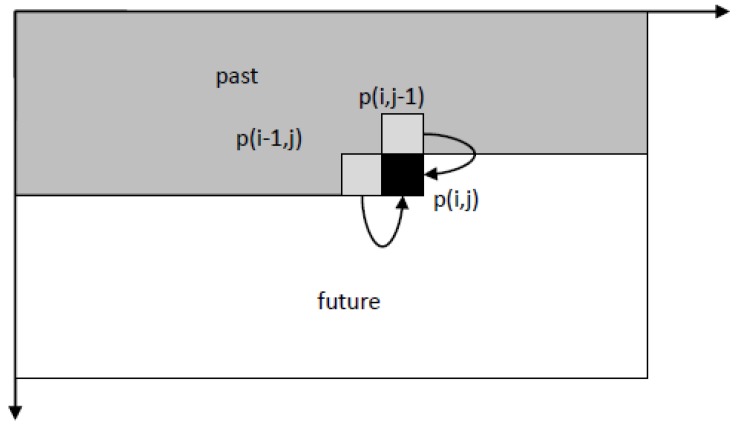
Operation sequence schematics of the 2D Kalman filter.

The Kalman filter operates recursively on streams of noisy input data to produce an estimator that is considered statistically optimal. The filter algorithm works in a two-step process. The first is the prediction step, where the Kalman filter calculates the estimates for the current state variables, as well as their uncertainties. The second step takes the outcome of the observations and calculates a new state using them alongside the estimates produced in the first step with a weight estimate. This process translates to two main operations: filtering outliers and ambiguous observations, and smoothing the generated surface. The two-dimensional Kalman filter operates on two orthogonal axis, where each step in the process looks at a singular point *p*(*i*, *j*) on the grid with a known value (observation, point measurement), and calculates a new value—if required—using the already updated values of the two previous points {*p*(*i* − 1, *j*), *p*(*i*, *j* − 1)} alongside each axis. This is schematically depicted in Figure 2. Such that the following steps are implemented: (1) choosing boundary conditions; (2) declaring a state vector; (3) calculating the a-priori state vector; (4) determining the model accuracy; (5) updating the state vector; (6) detecting outliers; and, (7) smoothing the results.

#### 3.4.1. Boundary Conditions

**Figure 3:**
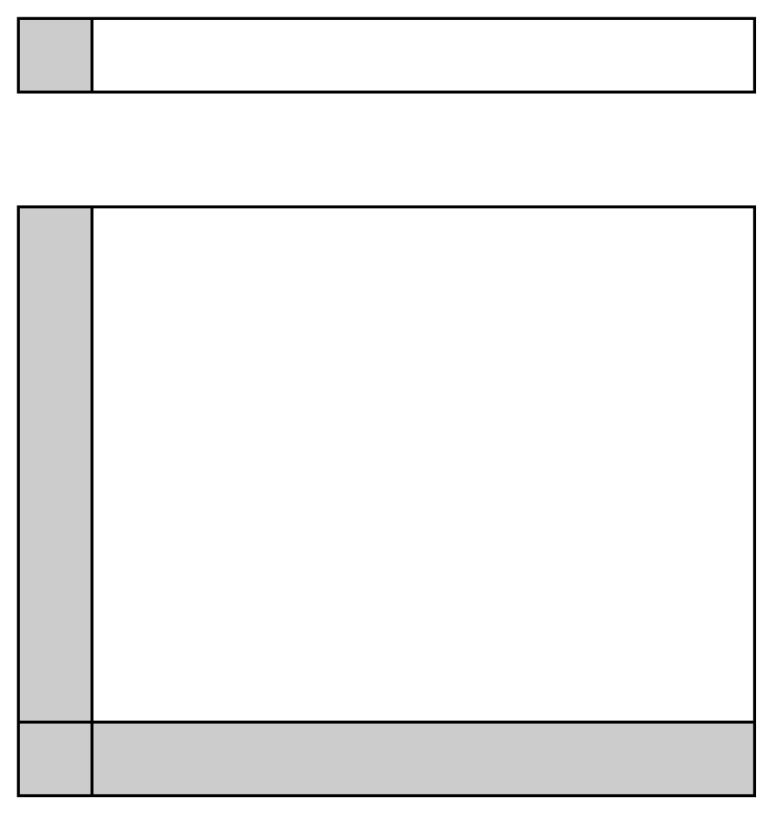
1D (**top**) and 2D (**bottom**) set of measurements (in white), and their corresponding boundary conditions (in grey).

A problem exists for the determination of the first sequence state; for this, a boundary condition must be used that will act as the first updated state. In the 2D Kalman filter, the boundary conditions are tied to both axes, which means that for the *X*-axis the entire first row acts as a set of boundary conditions and the same for the *Y*-axis. In fact, as depicted in Figure 3, one can see that the 2D Kalman filter requires a much larger set of boundary conditions than its 1D counterpart, and in many ways is more biased by these: the closer the measurement value is to the boundary conditions, the less effect the Kalman filter will have. This results in the fact that the Kalman filter operation improves the further it is from the process onset, e.g., the boundary.

#### 3.4.2. State Vector

A state vector representing each observation point in the grid must be defined. Here, each observation point is represented as a state vector, *S*, depicted in Equation (2), where (*i*, *j*) are observation point indices, *H*(*i*, *j*) is the observation point elevation, *H_x_*(*i*, *j*) is the first derivative along the *X*-axis, and *H_y_*(*i*, *j*) is the first derivative along the *Y*-axis:

(2) $$S(i,j)=\left[\begin{array}{c} H(i,j)\\ H_{x}(i,j)\\ H_{y}(i,j) \end{array}\right]$$

The state vector *S* can be further derived into two separate state vectors, *S_x_* and *S_y_*, depicted in Equation (3), one for each axis:

(3) $$\begin{array}{l} S_{x}(i,j)=\Phi_{x}(i,j)\cdot S(i-1,j)+V_{s_{x}}(i,j)\\ S_{y}(i,j)=\Phi_{y}(i,j)\cdot S(i,j-1)+V_{s_{y}}(i,j) \end{array}$$

where, $\Phi_{x}(i,j)=\left[\begin{array}{ccc} 1 & dx & 0\\ 0 & 1 & 0\\ 0 & 0 & 1 \end{array}\right]$, $\Phi_{y}(i,j)=\left[\begin{array}{ccc} 1 & 0 & dy\\ 0 & 1 & 0\\ 0 & 0 & 1 \end{array}\right]$, *V_sx_*(*i*, *j*) and *V_sy_*(*i*, *j*) are white noise vectors, and *dx* and *dy* are the grid resolutions in *X* and *Y*-axis, respectively.

#### 3.4.3. A-Priori State Vector

To calculate an a-priori state vector, “past” information must be known regarding the previously updated states alongside the *X* and *Y*-axis, respectively: *S*^+^(*i* − 1, *j*), *S*^+^(*i*, *j* − 1), and its co-variances *P*^+^(*i* − 1, *j*), *P*^+^(*i*, *j* −1). With this, the following can be calculated: (1) the a-priori state vectors *S*^−^*_x_*(*i*, *j*) and *S*^−^*_y_*(*i*, *j*) for each of the axis; (2) the a-priori state co-variance matrix *P*^−^*_x_*(*i*, *j*) and *P*^−^*_y_*(*i*, *j*) using a pre-determined model co-variance matrices, *Q_x_*(*i*, *j*) and *Q_y_*(*i*, *j*), respectively; and thus, (3) the a-priori state vector *S*^−^(*i*, *j*) with its co-variance matrix *P*^−^(*i*, *j*), depicted in Equation (4).

(4) $$\begin{array}{l} S_{x}^{-}(i,j)=\Phi_{x}(i,j)\cdot S^{+}(i-1,j)\\ S_{y}^{-}(i,j)=\Phi_{y}(i,j)\cdot S^{+}(i,j-1)\\ P_{x}^{-}(i,j)=\Phi_{x}(i,j)\cdot P^{+}(i-1,j)\cdot \Phi_{x}(i,j{)}^{T}+Q_{x}(i,j)\\ P_{y}^{-}(i,j)=\Phi_{y}(i,j)\cdot P^{+}(i,j-1)\cdot \Phi_{y}(i,j{)}^{T}+Q_{y}(i,j)\\ P^{-}(i,j)={\left(P_{x}^{-}{\left(i,j\right)}^{-1}+P_{y}^{-}(i,j{)}^{-1}\right)}^{-1}\\ S^{-}(i,j)=P^{-}(i,j)\cdot \left(P_{x}^{-}{\left(i,j\right)}^{-1}\cdot S_{x}^{-}(i,j)+P_{y}^{-}{\left(i,j\right)}^{-1}\cdot S_{y}^{-}(i,j)\right) \end{array}$$

#### 3.4.4. Model’s Accuracy

In the previous section, two matrices, *Q_x_*(*i*, *j*) and *Q_y_*(*i*, *j*), were included. These are the co-variances matrices of the model used in the Kalman filter process, representing the estimated accuracy of the model. The model used here relies on the Euler method, and thus, its accuracy can be determined accordingly. The accuracy estimation is depicted in Equation (5) for the *X*-axis, and in Equation (6) for the *Y*-axis:

(5) $$\begin{array}{l} v_{H_{b}}(i,j)\approx \frac{1}{2}H_{xx}(i-1,j)(dx{)}^{2}\\ v_{H_{xb}}(i,j)\approx H_{xx}(i-1,j)dx\\ v_{H_{yb}}(i,j)\approx H_{xy}(i-1,j)dx \end{array}$$

(6) $$\begin{array}{l} v_{H_{c}}(i,j)\approx \frac{1}{2}H_{yy}(i,j-1)(dy{)}^{2}\\ v_{H_{xc}}(i,j)\approx H_{yx}(i,j-1)dy\\ v_{H_{yc}}(i,j)\approx H_{yy}(i,j-1)dy \end{array}$$

where *H_xx_* is the second derivative alongside the *X*-axis, *H_yy_* is the second derivative alongside the *Y*-axis, *H_xy_* and *H_yx_* are the second derivatives alongside both axes, *dx* and *dy* are the resolutions alongside *X* and *Y*-axis, respectively, and *b* and *c* represent the (*i* − 1) and (*j* − 1) point heights, respectively. These equations show that the accuracy of the model is determined by the estimated accuracy of the second derivatives and the grid resolution. Thus, for example, if the second derivative accuracy is estimated at 0.08 (1/m) and the grid resolution is 5 m, then the estimated accuracy will be 1 m, while the X and Y derivatives estimated accuracies will be 0.4 (1/m). The equations also show that the higher the grid resolution is (i.e., smaller interval), the more accurate the model becomes when using the Kalman filter.

#### 3.4.5. Updated State Vector

After calculating the a-priori state vector and its covariance matrix, an updated estimated state vector (the a-posteriori state vector) can be calculated, depicted in Equation (7).

(7) $$\begin{array}{l} K=P^{-}(i,j)\cdot D^{T}\cdot {\left(D\cdot P^{-}(i,j)\cdot D^{T}+R\right)}^{-1}\\ S^{+}(i,j)=S^{-}(i,j)+K\cdot \left(Z-D\cdot S^{-}(i,j)\right)\\ P^{+}(i,j)=\left(I-K\cdot D\right)\cdot P^{-}(i,j) \end{array}$$

where *K* is the Kalman gain, *Z* is the measured point elevation, *R* is the known co-variance of *Z*, *D* is a [1 0 0] vector, *S*^+^(*i*, *j*) is the updated state vector, and *P*^+^(*i*, *j*) is the covariance of the updated state vector.

#### 3.4.6. Outlier Detection

Alongside the filter, an outlier detection operation is implemented, or otherwise, results might be skewed and coarse. This occurs when a gross error is introduced during the data acquisition process, or during the grid construction process, with the former being the most likely culprit, requiring data-filtering and smoothing. Thus, for each observation point within the grid, the difference between the a-priori state *H*^−^(*i*, *j*) and the measurement *Z*(*i*, *j*) are calculated, depicted in Equation (8).

(8) $$L(i,j)=z(i,j)-H^{-}(i,j)$$

In addition, a statistic measure *σ*_L(*i*,*j*)_ is calculated from the elevation accuracy of the model *σ*^2^*_H_*^−^_(*i*,*j*)_ and the measurement accuracy *R*(*i*, *j*), depicted in Equation (9).

(9) $$\sigma_{L(i,j)}=\sqrt{\sigma^{2}{}_{H^{-}(i,j)}+R(i,j)}$$

Based on Equations (8) and (9), a statistical test is performed using the critical value *ξ_α_*, depicted in Equation (10). If the test result is true, then only the model result value (the a-priori state vector) will be used, and if the result is false, the updated state vector result value will be used. This value is determined depending on the value of α (normal distribution); for example, if *α* is 0.05 (5% significant level) then *ξ_α_* will be 1.96, and if *α* is 0.01 then *ξ_α_* will be 2.58, and so forth.

(10) $$\left|L(i,j)\right|>\xi_{\alpha }\sigma_{L(i,j)}$$

#### 3.4.7. Smoothing

**Figure 4:**
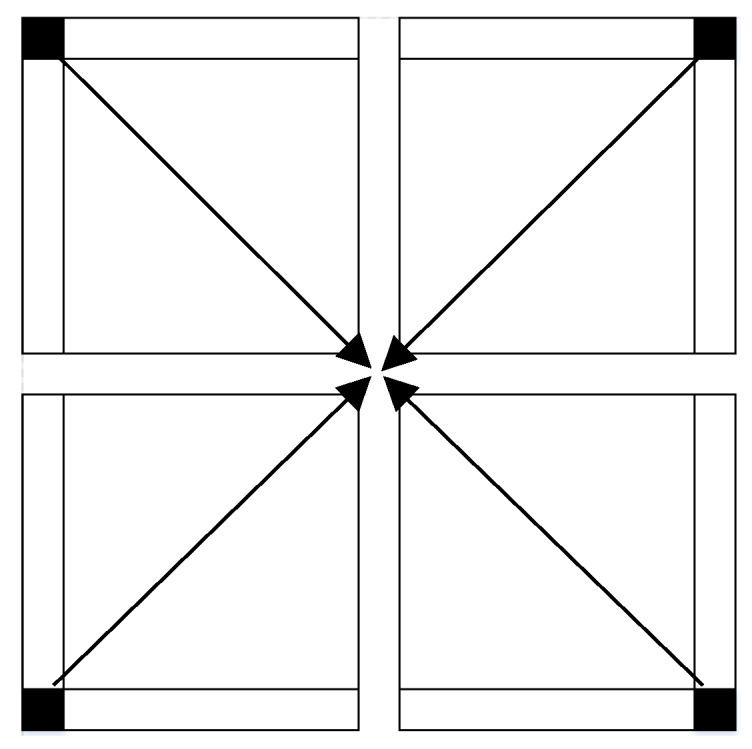
The four simultaneous Kalman filter processes implemented under four different boundary conditions.

Kalman filter results are inherently less accurate the closer they are to the boundary. As such, the Kalman filter process is executed simultaneously from four different directions, as depicted in Figure 4, allowing to use all grid boundaries as boundary conditions. Accordingly, four sets of surfaces are obtained, while for the final elevation determination an average between all is computed, resulting in a much smoother representation around the grid boundaries, while almost not affecting elevation values that are further from the boundaries.

### 3.5. Elevation Conversion

Since the reference DTMs used here for the evaluation of the accuracy analysis of the results represent geoid elevation, undulation conversion of the produced DTMs is implemented. The Israeli national undulation model is used (Israeli National Undulation Model—ILUM version 1.2), derived from 684 benchmark points using Kriging interpolation, resulting in a 0.5 km resolution grid. The average accuracy of the current version of this model is less than 10 m [35], with undulation values in the range of 19 and 21 m. For the area analyzed (The Technion, North of Israel), the value of 20.5 m was used. In addition, a supplementary 1 m reduction to elevation values was made, to compensate for the position of the smartphones during observations, which was held at waist height or on the car dashboard, in respect to the ground.

## 4. Experimental Results

**Figure 5:**
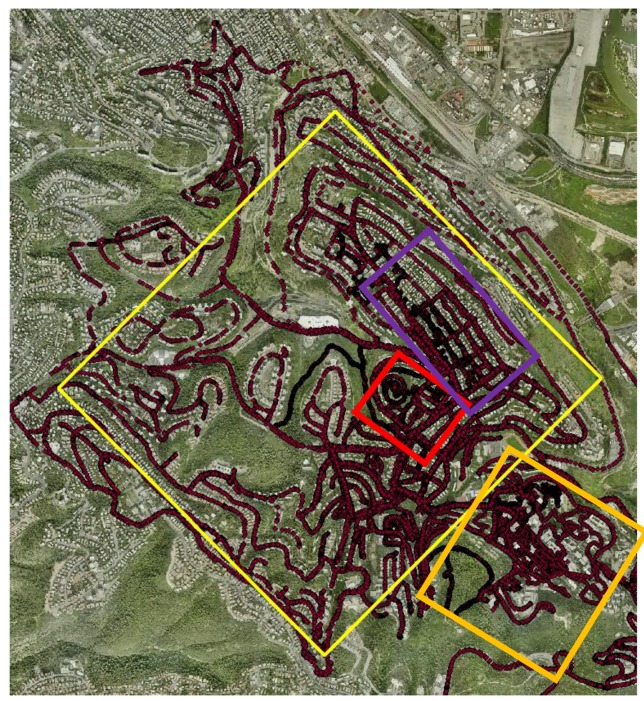
The area analyzed with the complete observation database (red dots). Purple depicts the residential area, red depicts the open area, orange depicts the Technion area, and yellow depicts the diverse area.

To imitate crowdsourcing with heterogeneous observations, various field campaigns were made near The Technion Campus, Haifa, Israel. Users were asked to collect data on a regular basis while walking and driving in the area, and from/to lessons/home. The idea is that even if users normally show recurrent routines visiting certain places, spending most of the time on a limited number of locations, the aggregation of all the data (and hence—crowdsourcing) will deliver a comprehensive representation of all the existing users’ visits in space. This methodology might suffer when locations are inaccessible, resulting in areas that might suffer from a low number of observations; the propagation of this effect is later investigated. Campaigns were made during a period of several days in June 2017, meaning that skies were mostly clear. Data from all campaigns were aggregated to form an A-GPS ground observation database—totaling in over 35,000 point observations, covering roughly 6.5 sq. km, depicted in Figure 5. All trajectories were collected via the various smartphone models (Figure 1) with a time interval of 1 s. The collected raw data was used to produce several DTMs: several covering relatively large heterogeneous areas and several covering relatively small more homogenous areas; all are depicted as rectangles in Figure 5. This was done to analyze the different data characteristics and the different existing environmental features of the area (e.g., different terrain types and data-collection means).

Emphasis was given to these data characteristics and environmental features for choosing and analyzing the different areas:

1. Environmental diversity—the idea is to collect data from different areas composed of diverse environmental settings. These include roads, walking paths, open areas, concealed areas near buildings and trees. The rationale is that these might have a different effect on the reliability of the observations (e.g., multipath and occlusions), thus allowing a broader examination and evaluation. Accordingly, collection means, i.e., walking and driving, might also have an effect.
2. Organization of observations—different areas impose constraints on the ability to collect field data and thus presenting varying point densities. Built areas, for example, are relatively uniform and homogenous in structure with building arrangements, thus allowing the collection of more ordered and controlled observations. This is opposite to open areas, mainly around woodland and extreme topography, which are harder to access, relying only on sparse paths, thus presenting more heterogeneous observation density and data-holes. Since interpolation is implemented on the raw observation data to generate a grid, this factor is important to analyze.

The observations’ elevation accuracy is set to 10 m; this value is chosen since it correlates to the medium to low-accuracy level of the existing A-GPS accuracies of all smartphone models used in the experiments (see Figure 1). Using this value allows facilitating a more comprehensive testing of the Kalman filter ability to cope with inaccurate estimates. The second derivative accuracies were set to 0.08 (1/m); this value was used since the analyzed topographies were mostly not rugged. However, it is very hard to determine the actual model accuracy, and in many cases, it can only be determined using an empirical practice, which was not the case here. Additionally, boundary conditions are set as follows: elevation value is equal to the value of the corresponding grid elevation value, while X and Y first derivative values are set to 0 (-); it was found that both these values have a minimal effect on the obtained results. The implementation was done using Matlab R2017b on an Intel i7-4790 CPU 3.6 GHz with 16 GB memory.

Two reference DTMs were used for statistical evaluation: (1) an authoritative DTM of Haifa, created on July 2014, based on aerial photogrammetry observations, having a planar resolution of 5 m and a vertical accuracy of 1 m; (2) an SRTM DTM covering Israel, with a resolution of 30 m, and an approximated vertical accuracy of 10 m. Regardless of the data acquisition technique used, errors will always be present [32], which are attributed to inaccuracies in the equipment, human errors, and errors related to transformation and interpolation. These propagate to three error types: random, systematic, and gross (blunders). To quantify the error, two descriptive statistics are used: MAD (Mean Absolute Deviation) representing the average absolute difference between the reference value and the produced DTM one, indicating how similar both DTMs are, proven to serve as a good measure of the absolute accuracy [36]; and, STDEV (STandard DEViation) representing the amount of variation within the DTMs (considered as the relative accuracy), indicating how dispersed the values are from the mean value [37]. In addition, since the height difference data did not have a normal distribution (according to the Jarque–Bera test), the Wilcoxon signed-rank test was implemented to evaluate the significance of the improvement [38], with an *α* value of 0.05 (5% significant level). This is a non-parametric statistical null hypothesis test that is used to compare two related datasets to evaluate whether their mean values differ, i.e., how significant is the improvement of the two datasets—before and after implementing the Kalman filter.

### 4.1. Residential Area

**Figure 6:**
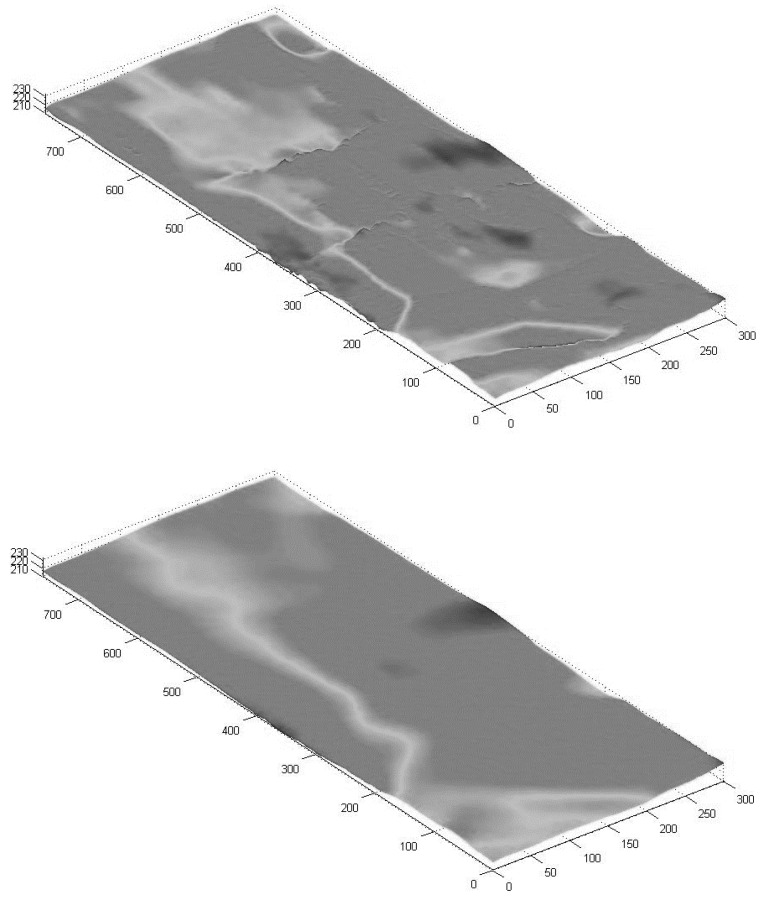
3D visualization of the 10 m resolution DTM for the residential area: pre-Kalman filter (**top**), and post-Kalman filter (**bottom**) (all values in meters).

The residential area (depicted in purple in Figure 5), covering close to 240,000 sq. m., have streets that are arranged in a grid shape, with a surface that is mostly flat. A total of 1428 observation points were collected mainly via walking trajectories, used to generate a 10 m IDW-based grid. The surface generated by the Kalman filter, depicted in Figure 6 (bottom), is smooth. This contrasts with the pre-Kalman filter (IDW-based) surface, which is more rugged and noisy, related to observation noise and gross errors artifacts exist in the raw data (Figure 6, top). The most notable noise areas are found in the center, most likely because these areas are interpolated data-holes and contain multipath errors (from the surrounding buildings). An analysis of the four-direction Kalman filter DTMs did indicate that the areas near the boundary conditions tend to be noisier than the areas farther away, although as expected, the resulting generated mean surface presented smooth and continuous representation around all edges.

The statistical evaluation results for the post-Kalman DTM, depicted in Table 1, show an improvement in all statistics. The MAD value is smaller than 2.50 m, proving that the produced surface is accurate even in an area that is affected from a relatively large volume of multipath errors and satellite obstructions caused by the nearby buildings and trees, as well as data-holes, which mostly affect the interpolation certainty. The STDEV value is less than 2 m, suggesting a high relative accuracy derived from consistent and precise observations of the A-GPS—well under the value of the expected 10 m. The pre-Kalman DTM also presented good statistical values, although the surface is more rugged with local topographic discontinuities, proving that A-GPS observations have the capacity to be reliable. We assume that the fact that most observations were collected while walking, resulting in a relatively homogenous data-density for the area, and the fact that the observed area is relatively flat (~20 m height change), contributed to the overall very good results of the produced DTM. The resulting *Z* value of the Wilcoxon signed-rank test was −4.7535, where the *h* value of *α* = 0.05 was 1, meaning that the null hypothesis is not rejected, i.e., there is no significant improvement when comparing the two surfaces.

### 4.2. Open Area

This area (depicted in red in Figure 5), covering close to 100,000 sq. m., contains a hill in the center surrounded by several streets with buildings. The hill itself is an open area with mostly clear skies with the occasional thicket area. Due to the nature of the area, a large part of it could not be surveyed since it is inaccessible by car and by foot, which leaves many data-holes. Total of 801 observation points was measured mostly via walking. The grid constructed contain points that were calculated using extrapolation, which reduces the overall accuracy of the DTMs, especially in the south-west region. All statistics, depicted in Table 2, show an improvement when the pre- and post-Kalman filter surfaces are compared to the reference DTM, mainly for the maximum difference. Both MAD and STDEV values are inferior to the residential area, though still under 5 m. Due to the fact that large portions of the boundary conditions use values that are based on extrapolation points, elevation bias is introduced into the Kalman filter process, which is manifested mainly in the maximum difference value, which is more than two times larger than of the previous area. This is definitely the main issue of using the 2D Kalman filter for DTM smoothing; however, the results show that if the bias is not too severe, the solution will not skew the results significantly, as they are still qualitative, where the implementation of the Kalman filter manages to improve the original raw results. The resulting *Z* value of the Wilcoxon signed-rank test was −0.6356, where the *h* value of *α* = 0.05 was 0, meaning that the null hypothesis is rejected, i.e., there is a significant improvement when comparing the two surfaces.

### 4.3. Technion Area

**Figure 7:**
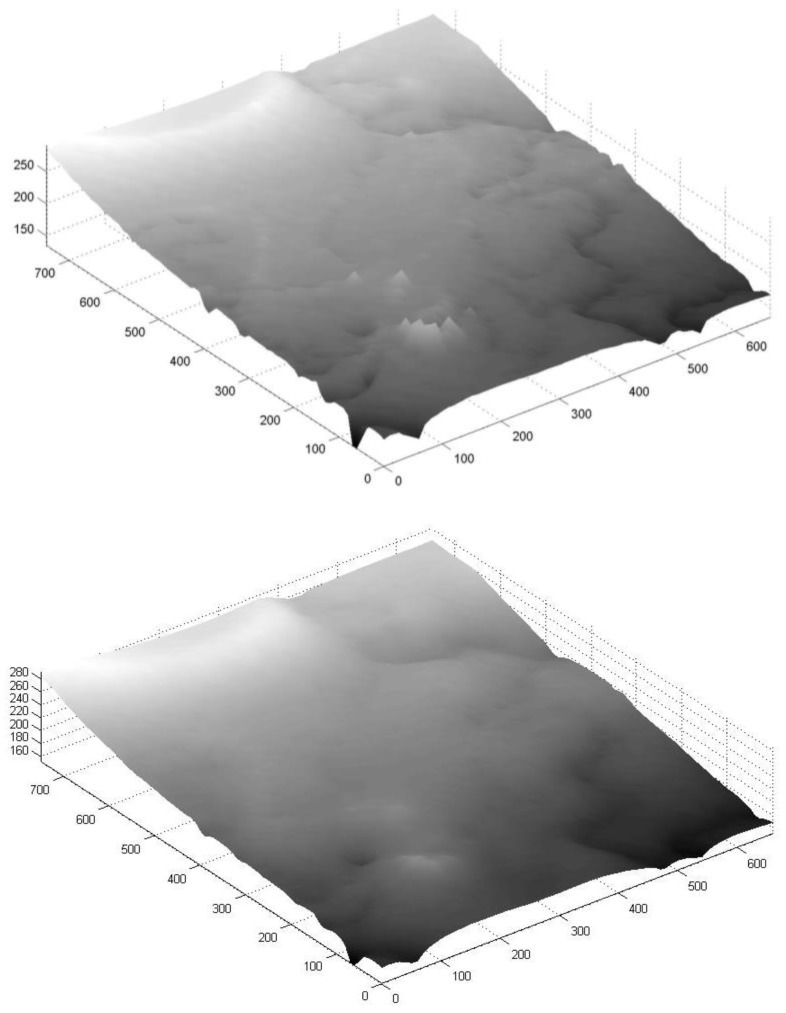
3D visualization of the 10 m resolution DTM for Technion area: pre-Kalman filter (**top**), and post-Kalman filter (**bottom**) (all values in meters).

The Technion area (depicted in orange in Figure 5) is very diverse: its topography is presented both with open areas, forested areas, residential areas, and combinations of all the above. Due to its varied characteristics and relatively small area (close to 500,000 sq. m with a height change of close to 100 m), it is potentially a very good testing area to evaluate the proposed implementation. A total of close to 4,200 observation points were collected, mostly via walking, resulting in a 10 m IDW-based grid with more than 5,000 points, depicted in Figure 7 (top). As expected, the noisiest areas are around the edges, with some notable spikes; erroneous areas are clearly visible that cannot represent the existing topography. The 2D Kalman filter managed to reduce most of these errors, and smooth the surface, with the resulting topography depicted in Figure 7 (bottom). The statistics for the raw and smoothed DTMs are depicted in Table 3, showing that the overall MAD and STDEV values are relatively small, even for the raw data, with a small improvement for both values after applying the Kalman filter. The maximum difference, however, was significantly decreased by more than 50%. The overall results of this area are promising, mainly because all observations were collected via non-authoritative instruments not requiring complex and costly processes to handle the data, and the fact that the area shows significant topographic variations. To further validate the results, the SRTM DTM of the area was analyzed with respect to the authoritative reference DTM. The statistical evaluation results prove that both DTMs present similar qualities for all statistics, although it should be noted that the post-Kalman DTM presents a larger resolution: 10 times more the number of SRTM points. The resulting *Z* value of the Wilcoxon signed-rank test was −3.7421, where the *h* value of *α* = 0.05 was 1, meaning that the null hypothesis is not rejected, i.e., there is no significant improvement when comparing the two surfaces.

### 4.4. Diverse Large Area

**Figure 8:**
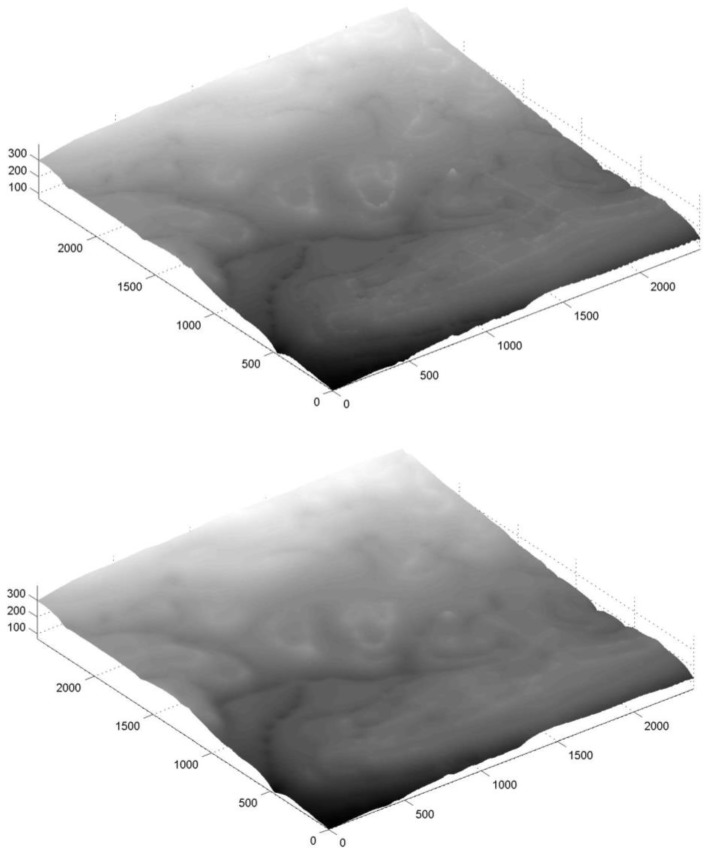
3D visualization of the 10 m resolution DTM for the entire area: pre-Kalman filter (**top**), and post-Kalman filter (**bottom**) (all values in meters).

The majority of the data collected during the field surveys was used, totaling in over 25,000 observation points covering 6.5 sq. km. This analysis simulates the use of crowdsourced heterogeneous data from A-GPS trajectories for various areas, having various data characteristics, environmental features, and collection means (here, most trajectories were collected via cars, which, at least theoretically, show lower quality). The resulting 10 m resolution DTM contains 60,000 points. Both pre- and post-Kalman filter surfaces are depicted in Figure 8, with the statistical results depicted in Table 4. The statistical values are bigger than the ones retrieved for the other small areas. This is mainly derived from the fact that this area presented relatively large data holes, sometimes close to 500 m in diameter; the farther the points calculated via the IDW interpolation are from existing observations, they introduce more noise and error into the generated grid. Such that there exists a strong correlation between the magnitude of the elevation differences (also associated with areas having low observation density) and the resulting quality of that area. Also, topography influences the different statistical values, which coincides with the fact that the height values are calculated based on a model. Moreover, it is no surprise that the larger the area is, more observation errors can occur, some of which are blunders, which are introduced into the statistical evaluation. Results show that the 2D Kalman filter is effective in filtering and smoothing the DTM, proving its ability to operate on larger scale datasets, which combine flat zones, mountainous areas, built areas, concealed (vegetation) areas and different collection means. The Kalman filter managed to minimize the maximum difference value in more than 30%, slightly reducing the overall MAD and STDEV values. Similar to the Technion area, the SRTM DTM of the area was evaluated with respect to the authoritative reference DTM. The statistical evaluation results show that the produced DTM and the SRTM present similar qualities, whereas the SRTM is slightly more qualitative than the produced DTM. When the elevation difference values are analyzed in respect to the authoritative reference DTM, most of the elevation difference values are small: 40% are less than 5 m, and 68% are less than 10 m—values that validate the good accuracy level of the A-GPS elevation observations. The resulting *Z* value of the Wilcoxon signed-rank test was −7.3132, where the *h* value of *α* = 0.05 was 1, meaning that the null hypothesis is not rejected, i.e., there is no significant improvement when comparing the two surfaces.

### 4.5. Summary

Based on the statistical analyses, it appears that for all scenarios and datasets, regardless of the environmental settings and conditions, density and acquisition means, the 2D Kalman filter produces good surface results. When compared to an accurate DTM, the small areas produced very good MAD and STDEV values, which are less than 6 m, sometimes even less than 3 m, with the maximum difference that is significantly reduced. For the large area, both MAD and STDEV values are around 8 m, which is also very promising. When compared to SRTM DTM of the area, all crowdsourced A-GPS DTMs show slightly inferior to similar qualities, which demonstrate the potential of using crowdsourcing of A-GPS smartphone observations to produce DTMs. It should be noted that for the areas the SRTM is analyzed, A-GPS DTMs show a much higher volume of DTM points, which might influence the resulting statistical values. As expected, data acquisition in open areas produce less accurate results, mainly due to sparse data density that is the result of data collection constraints, which affects the results and reliability of the IDW interpolation, and thus of the surface constructed. Smooth areas, on the other hand, show very promising results, as well as areas having large volumes of observations, such as the Technion area, which further validates the use of the crowdsourcing methodology. It appears that data acquired via walking is more accurate than driving, although this requires additional analysis. An interesting issue is that although the MAD value varies, the STDEV value is mostly consistent and low—around 1.5–4.5 m (for the small areas), suggesting that both the raw observations and the generated surfaces are precise and have a high inner accuracy.

The Wilcoxon signed-rank test showed that for most areas there was no significant improvement of the post-Kalman surface, which corresponds to the relatively small improvement values of the MAD and the STDEV values. Still, for all areas the implementation of the 2D Kalman filter did improve the overall results, filtering errors and smoothing the raw surfaces, also producing topographies that are in line with the SRTM quality.

## 5. Conclusions and Future Work

Although current research prove that the accuracy of A-GPS measurements continues to improve [3], with a wide range of studies to support this conception by utilizing this for a variety of ubiquitous applications and services, ranging from transportation (e.g., [20,21]) to health services [18], still most focus on the use of the horizontal data only. To provide with a more comprehensive solution, this research paper emphasis is given to the combination of all three dimensions, with the specific customized handling of the vertical one. Thus, extending the current state of the art by presenting the feasibility of producing qualitative DTM infrastructures from massive crowdsourced ubiquitous A-GPS trajectories collected with off-the-shelf mobile smartphones. The main issue here is related to the heterogeneity and uncertainty of the accumulated data, due to the way user-generated data is usually collected—by non-professional sources using somewhat inaccurate observation tools. Experiments and analyses carried out proved that the implementation of the developed 2D Kalman filter algorithm is robust. The Kalman filter allows more dynamization in the process, and unlike other common filters (e.g., Low Pass filter), it considers not only the local area during the process, but it also uses the process history to provide the estimation.

DTMs produced were found to have similar qualities when compared to public domain DTM sources, such as the SRTM, while offering much higher data-density. Moreover, the crowdsourced ubiquitous methodology offers a much cheaper and faster production process, suggesting the possibility to replace SRTM as the topographic infrastructure for open source mapping infrastructures, such as OSM. When compared to an authoritative DTM, the produced DTMs were found to be comparatively accurate and reliable with MAD and STDEV values that are lower than 8 m for all areas analyzed, where small areas showed better values, even where data-holes exist due to areas that are inaccessible. Outlier removal produced very good results, where for some areas the maximum errors were reduced by more than 50%. It is interesting to note that the results were almost not affected by the volume of data. This fact is in line with our assumption that the Kalman filter tends to perform better while improving its model and prediction over time. Another key element is the fast computing time, which even for the largest area was less than 10 s.

Future work is planned for improving the Kalman filter algorithm, e.g., reducing the effect of the boundary conditions by implementing different techniques for averaging, and improving the model’s accuracy by using co-variance values calculated by the individual devices during the field experiments. Future research will try to combine the generated DTM with existing free-to-use sources, such as SRTM, to improve their resolution and overall accuracy, mainly in urban areas where large volumes of crowdsourced user-generated position and elevation data exists. Overall, the results presented here are very promising and qualitative, while having the potential to contribute to open-source mapping infrastructures that are widely available and used today.

## Figures and Tables

**Table 1:** Statistical evaluation of elevation differences in respect to the photogrammetric reference DTM: 10 m pre- and post-Kalman filter DTM (2356 grid-points).

	Pre-Kalman Filter	Post-Kalman Filter	Improvement
Abs Max Difference	10.3 m	7.9 m	23%
MAD	2.6 m	2.4 m	8%
STDEV	1.9 m	1.7 m	11%

**Table 2:** Statistical evaluation of elevation differences in respect to the photogrammetric reference DTM: 10 m pre- and post-Kalman filter DTM (1056 grid-points).

	Pre-Kalman Filter	Post-Kalman Filter	Improvement
Abs Max Difference	23.1 m	17.1 m	26%
MAD	5.1 m	4.6 m	10%
STDEV	4.2 m	3.5 m	17%

**Table 3:** Statistical evaluation of elevation differences in respect to the photogrammetric reference DTM: 10 m pre- and post-Kalman filter DTM (5092 grid-points) and 30 m SRTM (575 grid-points).

	Pre-Kalman Filter	Post-Kalman Filter	Improvement	SRTM
Abs Max Difference	55.0 m	25.5 m	55%	33.4 m
MAD	6.4 m	6.3 m	2%	5.1 m
STDEV	5.1 m	4.5 m	12%	3.5 m

**Table 4:** Statistical evaluation of elevation differences in respect to the photogrammetric reference DTM: 10 m pre- and post-Kalman filter DTM (60,000 grid-points) and SRTM (7200 points).

	Pre-Kalman Filter	Post-Kalman Filter	Improvement	SRTM
Abs Max Difference	85.9 m	57.1 m	34%	47.6 m
MAD	8.7 m	8.6 m	1%	4.9 m
STDEV	7.8 m	7.7 m	1%	4.0 m
